# Renal C4d is a potential biomarker of disease activity and severity in pediatric lupus nephritis patients

**DOI:** 10.3389/fped.2023.1193917

**Published:** 2023-06-01

**Authors:** Xueyao Wang, Shaojie Fu, Jinyu Yu, Daru Tang, Hao Wu, Zhonggao Xu

**Affiliations:** ^1^ Department of Nephrology, The First Hospital of Jilin University, Changchun, China; ^2^ Department of Renal Pathology, The First Hospital of Jilin University, Changchun, China; ^3^ Medical Student, Bethune Medical College, Jilin University, Changchun, China

**Keywords:** C4d, pediatric lupus nephritis, biomarker, disease activity, peritubular capillaries

## Abstract

**Background:**

Systemic lupus erythematosus (SLE), a multisystemic autoimmune disease, is very aggressive in pediatric-onset patients as they are prone to develop lupus nephritis (LN). Although renal C4d positivity is correlated with the activity of renal disease and SLE in adult-onset LN patients, available information for pediatric-onset patients is limited.

**Methods:**

To evaluate the potential diagnostic significance of renal C4d staining in pediatric LN patients, we retrospectively detected C4d staining by immunohistochemistry on renal biopsy specimens from 58 pediatric LN patients. The clinical and laboratory data at the time of the kidney biopsy and the renal disease activity of histological injury were analyzed according to the C4d staining status.

**Results:**

Glomerular C4d (G-C4d)-positive staining was detected in all 58 cases of LN. Patients with a G-C4d score of 2 displayed more severe proteinuria than those with a G-C4d score of 1 (24-h urinary protein: 3.40 ± 3.55 g vs. 1.36 ± 1.24 g, *P *< 0.05). Peritubular capillary C4d (PTC-C4d) positivity was found in 34 of 58 LN patients (58.62%). The PTC-C4d-positive patient groups (patients with a PTC-C4d score of 1 or 2) had higher serum creatinine and blood urea nitrogen levels as well as renal pathological activity index (AI) and SLE disease activity index (SLEDAI) scores; however, they had lower serum complement C3 and C4 levels compared to PTC-C4d-negative patients (*P < *0.05). In addition, there was positive tubular basement membrane C4d (TBM-C4d) staining in 11 of 58 LN patients (18.96%), and a higher proportion of TBM-C4d-positive patients than TBM-C4d-negative patients (63.63% vs. 21.27%) had hypertension.

**Conclusion:**

Our study revealed that G-C4d, PTC-C4d, and TMB-C4d were positively correlated with proteinuria, disease activity and severity, and hypertension, respectively, in pediatric LN patients. These data suggest that renal C4d is a potential biomarker for disease activity and severity in pediatric LN patients, providing insights into the development of novel identification and therapeutic approaches for pediatric-onset SLE with LN.

## Introduction

Systemic lupus erythematosus (SLE) is a chronic, multisystemic autoimmune disease. Even though SLE can occur at any age, 15%–20% of all lupus cases begin in childhood (1, 2). Pediatric-onset SLE patients have a more aggressive disease course than adult-onset SLE patients, and their disease is more frequently complicated with lupus nephritis (LN) (3, 4). It has been reported that approximately 50%–82% of pediatric SLE patients have histologically confirmed LN, compared to 20%–40% of adult SLE patients (5, 6). The diagnosis and severity of renal illness are crucial, since the therapeutic options are frequently determined by the degree of kidney involvement (7). Therefore, the discovery of a diagnostic biomarker for pediatric LN is urgently needed.

C4d is becoming more widely acknowledged as a potential biomarker in several diseases where antibodies can cause tissue damage, since positive detection of the complement split product C4d along peritubular capillaries (PTCs) in renal allograft specimens as a biomarker for antibody-mediated rejection was introduced in routine clinical practice in the late 1990s (8–11). Generated by C4 activation in both the classical pathway and the lectin pathway, C4d maintains covalent bonds with tissue components, including endothelial surfaces and basement membranes, via a thiol-ester site (12, 13). Increasing evidence has shown that C4d-positive detection in renal biopsy specimens from LN patients correlates with the activity of renal disease and SLE, suggesting that renal C4d could be a potential indicator of disease activity in SLE patients with LN (14–16). Notably, the majority of current research on renal C4d detection in LN patients focuses on the cohort with adult-onset SLE. However, information regarding pediatric-onset SLE is still limited.

In this study, we explored renal C4d deposition in pediatric LN patients to evaluate its potential diagnostic value in pediatric-onset SLE with LN, taking into account the clinical significance of LN among pediatric SLE patients and its distinction from adult-onset SLE. In addition, the association of renal C4d staining with the disease activity and clinical manifestation of LN patients was analyzed. These data suggested that renal C4d is a potential biomarker of disease activity and severity in pediatric LN, shedding a light on the development of novel identification and treatment approaches for pediatric-onset SLE with LN.

## Material and methods

### Study design and patients

We retrospectively analyzed all pediatric LN patients who underwent renal biopsy at the Department of Pediatrics at the First Hospital of Jilin University from February 2016 to January 2021. Patients who met the following inclusion criteria were included: (1) aged <18 years old at the time of the renal biopsy; (2) the specimen size was adequate for light microscopy (≥10 glomeruli); (3) there were enough clinical and laboratory data available at the time of the renal biopsy. Demographics, renal function, proteinuria, hematuria, and serologies for lupus, etc. were among the clinical and laboratory data. A total of 58 patients were included. All patients fulﬁlled the American College of Rheumatology classiﬁcation criteria for the diagnosis of SLE (17). Global disease activity was calculated using the systemic lupus erythematosus disease activity index (SLEDAI) (18). LN was confirmed in all patients by renal biopsy. Other renal disease control samples were obtained from 10 individuals with minimal change disease (MCD), 10 with IgA nephropathy (IgAN) and 5 with membranous nephropathy (MN). Specimens were obtained and used for this study according to the ethical guidelines established by Jilin University and its affiliated hospitals.

### Histological examinations of renal biopsies

The diagnosis of LN was based on histological assessment of renal biopsy paraffin-embedded tissue sections stained with hematoxylin and eosin, Masson's trichrome, periodic acid-Schiff, and methenamine silver for light microscopy. Small portions of freshly frozen renal sections were stained with ﬂuorescein isothiocyanate- conjugated rabbit antisera against human IgG, IgA, IgM, C1q, C3, and fibrinogen (Dako, Denmark) by direct immunoﬂuorescence. The International Society of Nephrology/Renal Pathology Society (ISN/RPS) 2003 classiﬁcation of LN was used to categorize the biopsy specimens (19). According to the methods reported by Austin et al., the pathological activity index (AI) and chronicity index (CI) of renal biopsies indicating LN were calculated (20).

### Immunohistochemical examinations of renal C4d

Formalin-fixed, paraffin-embedded tissue sections were stained using the immunoperoxidase method with rabbit anti-human C4d polyclonal antibody (clone BI-RC4D, Biomedica, GmbH). After 2-*μ*m-thick sections were deparaffinized and rehydrated, antigen retrieval was quickly performed using heat for 5 min in a Tris/EDTA buffer (pH 8.5), followed by 0.4% pepsin for 5 min at room temperature. The endogenous peroxidase activity was blocked with 3% H_2_O_2_. To prevent nonspecific labeling, 10% normal goat serum was employed. The primary antibody of C4d was applied at a dilution of 1:20 and incubated overnight at 4°C, which was followed by incubation with secondary antibodies (goat anti-mouse/rabbit) for 1 h at room temperature. The Max Vision-HRP Kit (Maixin Company, Fuzhou, China) and 3-amino-9-ethylcarbazole (AEC) were utilized for visualization. Slides were mounted with AEC Mounting Solution after being counterstained with hematoxylin. Renal biopsy tissue from allografts with antibody-mediated rejection was employed as the positive control, and the omission of the primary antibody was used as the negative control.

### Evaluation of C4d staining

Glomerular C4d (G-C4d) staining was scored semiquantitatively based on the intensity of the staining reaction. A score of 0 indicates negative staining (no glomerular staining), a score of 1 indicates weak-to-moderate glomerular staining, and a score of 2 indicates intense staining.

Peritubular capillary C4d (PTC-C4d) staining was scored semiquantitatively based on the percentage of PTC-C4d positivity present in the stained tissue by reference to the Banff Classification of Renal Allograft Pathology (21). A score of 0 indicates negative staining (absence of C4d staining in PTCs), a score of 1 indicates minimal-to-focal C4d staining (present in <25% of PTCs), and a score of 2 indicates multifocal-to-diffuse C4d staining (present in ≥25% of PTCs).

Tubular basement membrane C4d (TBM-C4d) staining was considered as positive when ≥5% of tissue was stained.

### Statistical analysis

GraphPad Prism 5 was utilized to perform all statistical analyses. One-way analysis of variance and the Mann–Whitney test were used to compare the continuous variables. To compare the frequency between multiple groups, Fisher's exact test and the chi-squared test were applied. A two-tailed *P < *0.05 was considered to be statistically significant.

## Results

### Clinical and pathological characteristics of pediatric LN patients

There were 58 pediatric LN patients enrolled in this study. Their clinical and pathological characteristics at the time of the renal biopsy are listed in Table 1. These patients, ranging in age from 4 to 16 years old (mean: 11.89 ± 2.66 years old), were more likely to be female (54 cases, 93.1%) than male. Though all patients (100%) were affected by proteinuria, only 25 cases (43.1%) were in the nephrotic range. Hematuria and impaired renal function were detected in 46 patients (79.31%) and 20 patients (44.44%), respectively. For the detection of serum complements, 56 cases (96.55%) and 52 cases (89.65%) had low C3 and C4 levels, respectively. Antinuclear antibodies were identified in the serum of all 58 LN patients (100%); and in 54 (93.1%) of these cases, anti-double-stranded DNA (anti-dsDNA) was also detected. In addition, 17 patients (29.31%) suffered from systemic hypertension (22). Moreover, the average SLEDAI value was 18.72 ± 5.21 (range: 4–28).

**Table 1:** Clinical data and pathological findings in 58 LN patients.

	LN patients (*n *= 58)
Sex (female/male)	54/4
Age (years, mean ± sd)	11.89 ± 2.66 (range: 4–16)
Nephrotic-range proteinuria (yes/no)	25/33
Hematuria (yes/no)	56/2
Impaired renal function (yes/no)	20/38
Low serum C3 (yes/no)	56/2
Low serum C4 (yes/no)	52/6
ANA (positive/negative)	58/0
Anti-dsDNA (positive/negative)	54/4
Hypertension (yes/no)^a^	17/41
SLEDAI (mean ± sd)	18.72 ± 5.21 (range: 4–28)
**Pathological ISN/RPS classification**	
I	0
II	1
III	6
IV	45
V	1
VI	0
III + V	2
IV + V	3
Activity Index (mean ± sd)	10.41 ± 3.91 (range: 1–19)
Chronicity Index (mean ± sd)	0.17 ± 0.77 (range: 0–5)

SLEDAI, systemic lupus erythematosus disease activity index; ANA, antinuclear antibodies; Anti-dsDNA, anti-double-stranded DNA antibody; mean ± sd: mean ± standard deviation.

^a^ Hypertension was defined as systolic or diastolic blood pressure above the 95th percentile for age, height, and sex (22).

Histologically, all 58 renal biopsy specimens showed significant staining of all three immunoglobulin classes (IgG, IgM, and IgA) as well as both complement components (C3 and C1q) on the glomeruli. Among the 58 patients, 1 was classified as class II, 6 as class III, 45 as class IV, 1 as class V, 2 as class III + V, 3 as class IV + V, and none as class I or class VI, according to the ISN/RPS classification. The overall mean renal pathological AI and mean CI were 10.41 ± 3.91 (range: 1–19) and 0.17 ± 0.77 (range: 0–5), respectively (Table 1). Of the 58 LN cases, 4 cases had active accompanying chronic injuries, while the other 54 cases did not. This study did not evaluate the CI because only four renal specimens revealed histological evidence of chronic damage.

### G-C4d was positively correlated with proteinuria in pediatric LN patients

**Figure 1:**
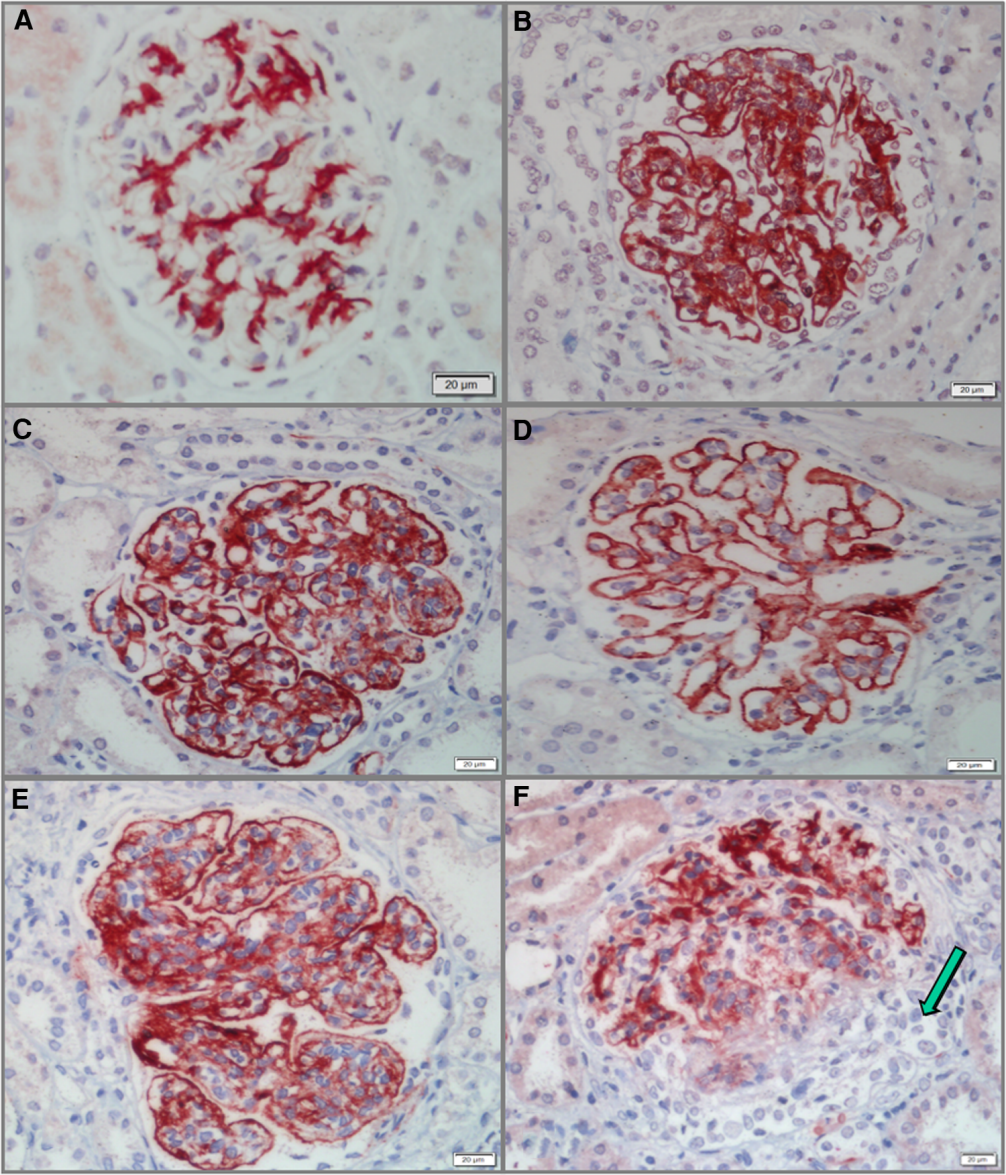
Glomerular C4d (G-C4d) staining in pediatric lupus nephritis (LN). (**A**) C4d staining was mainly confined to the mesangium in class II LN. (**B**,**C**) C4d granular staining in the capillary walls and to a greater or lesser extent in the mesangium in classes III (**B**) and IV (**C**). (**D**) In class V with concurrent class III, C4d staining consistently highlighted granular membranes. (**E**) C4d staining was heterogeneous in marked hypercellularity. (**F**) C4d staining was negative for crescent lesions (arrow). Bar = 20 µm.

**Figure 2:**
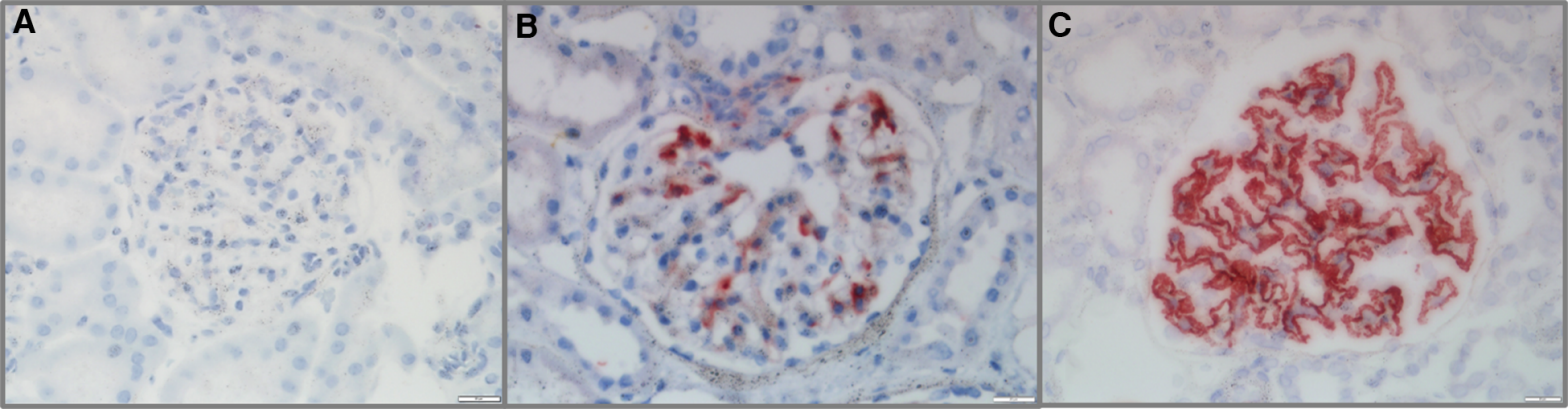
Glomerular C4d (G-C4d) staining in pediatric renal diseases. (**A**) No G-C4d staining was detected in minimal change disease. (**B**) In proportion of IgA nephropathy, C4d staining was mostly in the mesangial area. (**C**) C4d granular staining in the capillary walls of membranous nephropathy (Bar = 20 µm).

All 58 cases of pediatric LN were detected to have G-C4d-positive staining, most of which was located in the capillary walls of the glomeruli. Notably, the staining pattern was correlated with the pathological class. Class II showed staining that was restricted to the mesangium (Figure 1A), whereas classes III and IV displayed a mixture of granular or confluent staining in the peripheral capillary walls and mesangial staining with irregular distributions (Figures 1B,C). Granular membranous staining in class V cases was consistently highlighted by C4d, and this result did not depend on whether class III or IV was present concurrently or not (Figure 1D). In addition, C4d staining was not homogeneous in areas of substantial hypercellularity (Figure 1E) and was negative for crescent-shaped lesions (Figure 1F). Meanwhile, for other renal disease control samples, no G-C4d staining noted definitely in all 10 MCD cases (Figure 2A); 6 of 10 IgAN were detected mostly mesangial C4d deposition (Figure 2B); and C4d deposition was present in all 5 MN cases with granular membranous distribution (Figure 2C). These findings showed that the distribution of G-C4d positive staining and C4d deposit in the glomerulus are dependent on various renal disorders.

According to the G-C4d staining scores, 13 patients had a score of 1 (22.41%), while a score of 2 was observed in 45 LN patients (77.58%). No patients had G-C4d staining results that were negative. Clinically, patients with a G-C4d score of 2 displayed more severe proteinuria than those with a G-C4d score of 1 (Table 2; 24-h urinary protein: 3.40 ± 3.55 g vs. 1.36 ± 1.24 g, *P < *0.05). Meanwhile, comparison of the patients who had a G-C4d score of 1 vs. those with a score of 2 showed that the mean age, serum creatinine, blood urea nitrogen, hematuria, serum C3 and C4, as well as proportions of hypertension and serum anti-dsDNA detection were not significantly between the two groups of patients. Also, neither the SLEDAI nor the renal pathological AI was observed to be significantly correlated with the G-C4d staining score in the 58 pediatric LN patients (Table 2). Regarding different pathological classes of LN, no significant correlation between the LN class and the G-C4d staining score was found (Table 3). These data indicated that C4d, in glomeruli, is a histologic marker of complement activation and those cases showing more intense staining had more proteinuria.

**Table 2:** Clinical data and activity Index of renal injury according to the G-C4d staining scores and TBM-C4d staining, respectively.

Characteristic	G-C4d score			TBM-C4d		
	Score 1 (*n *= 13)	Score 2 (*n *= 45)	*P-*value	Negative (*n *= 47)	Positive (*n *= 11)	*P-*value
Age (years)	8.84 ± 3.18	10.88 ± 4.01	0.0977	11.82 ± 2.48	12.09 ± 3.67	0.7680
Proteinuria (g/24 h)	1.36 ± 1.24	3.40 ± 3.55	0.0476*	2.69 ± 2.65	3.96 ± 3.37	0.1799
Hematuria (RBC/HPF)	89.63 ± 132.86	173.48 ± 276.02	0.2957	153.66 ± 236.38	152.4 ± 231.83	0.9870
Serum creatinine (µmol/L)	57.55 ± 27.14	75.37 ± 39.08	0.1302	66.77 ± 31.03	90.97 ± 54.50	0.0517
Blood urea nitrogen (mmol/L)	8.15 ± 4.70	8.85 ± 4.79	0.6430	8.16 ± 4.10	10.95 ± 6.55	0.0776
Serum C3 (g/L)	0.468 ± 0.273	0.379 ± 0.260	0.2868	0.403 ± 0.221	0.381 ± 0.243	0.7721
Serum C4 (g/L)	0.061 ± 0.040	0.050 ± 0.027	0.2532	0.051 ± 0.027	0.061 ± 0.042	0.3276
Anti-dsDNA, *n* (%)	11 (84.61%)	43 (95.55)	0.214	44 (93.61%)	10 (90.90%)	1.00
Hypertension, *n* (%)	3 (23.07%)	14 (31.11%)	1.0	10 (21.27%)	7 (63.63%)	0.009^#^
SLEDAI	16.30 ± 6.61	19.42 ± 4.58	0.0563	18.48 ± 5.49	19.72 ± 3.82	0.4821
Activity index	8.84 ± 3.18	11.09 ± 3.86	0.0602	10.25 ± 3.87	11.18 ± 4.19	0.4827

Data are expressed as the mean ± standard deviation except for hypertension and anti-dsDNA. RBC/HPF: red blood cells/high-power field.

* *P < *0.05.

^#^ *P *< 0.01.

**Table 3:** G-C4d and PTC-C4d scores as well as TBM-C4d staining for different classifications of LN.

Pathological class (*n*)	G-C4d score			PTC-C4d score			TBM-C4d		
	Score 1 (*n *= 13)	Score 2 (*n *= 45)	*P-*value	Score 0 (*n *= 24)	Score 1 or 2 (*n *= 34)	*P-*value	Negative (*n *= 47)	Positive (*n *= 11)	*P-*value
II (1), *n* (%)	1 (7.69)	0		1 (4.16)	0		1 (2.12)	0	
III (6), *n* (%)	3 (23.07)	3 (6.66)	0.119	3 (12.50)	3 (8.823)	0.671	5 (10.6)	1 (9.09)	1.00
IV (45), *n* (%)	8 (61.53)	37 (82.22)	0.1407	15 (62.50)	30 (88.23)	0.027*	35 (74.46)	10 (90.9)	0.433
V (1), *n* (%)	0	1 (2.22)		1 (4.16)	0		1	0	
III + V (2), *n* (%)	0	2 (4.44)		2 (8.33)	0		2 (4.25)	0	
IV + V (3), *n* (%)	1 (7.69)	2 (4.44)		2 (8.33)	1 (2.94)		3 (6.38)	0	

* *P < *0.05.

### PTC-C4d correlates with global and renal disease activity in pediatric LN patients

**Figure 3:**
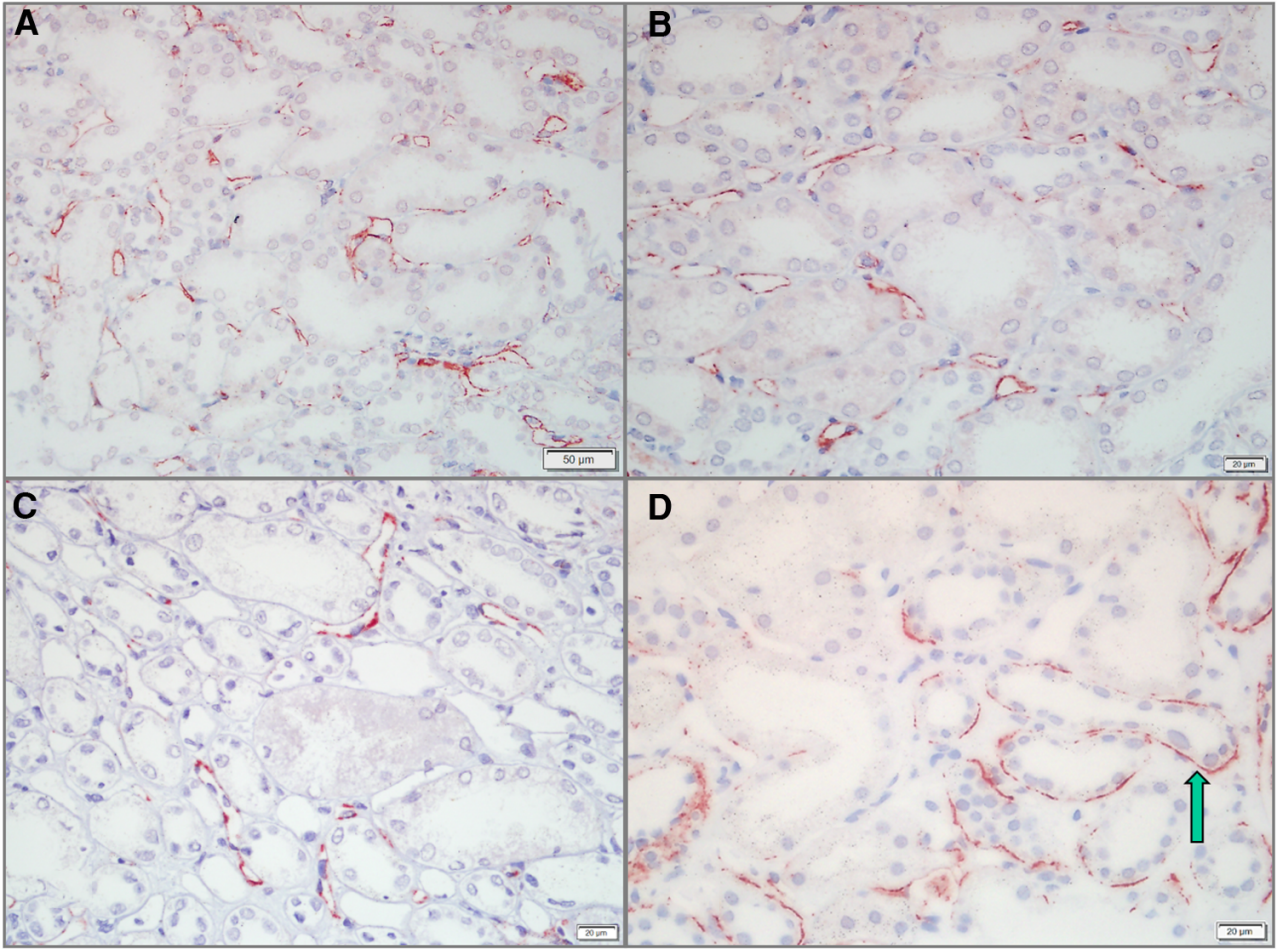
C4d staining along the peritubular capillaries (PTCs) and in the tubular basement membrane (TBM) of pediatric lupus nephritis (LN) samples. (**A,B**) C4d deposits along PTCs in LN cases. Staining was granular and showed a diffuse distribution in >25% of PTCs (Bar = 50 µm in **A**; Bar = 20 µm in **B**). (**C**) PTC-C4d staining was granular and focal along the PTCs, <25% of PTCs (Bar = 20 µm). (**D**) C4d deposits in the TBM of LN cases. C4d staining was tiny granular and showed a focal distribution (arrow) (Bar = 20 µm).

**Figure 4:**
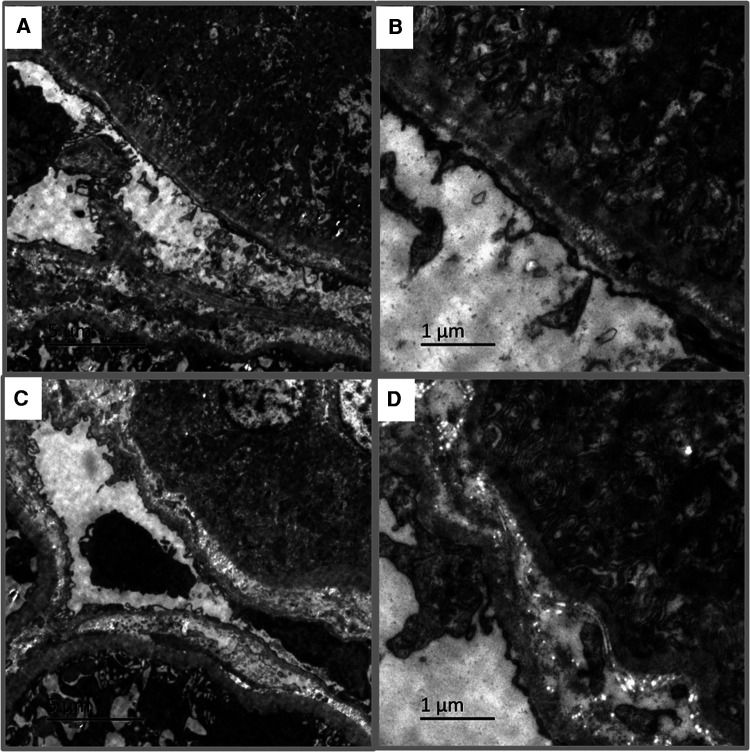
Transmission electron microscopy in pediatric lupus nephritis (LN). (**A,B**) Electron-dense deposits were not observed in peritubular capillaries (PTCs) area of PTC-C4d positive staining case (Bar = 5 µm in **A**; Bar = 1 µm in **B**). (**C,D**) No electron-dense deposits appeared in tubular basement membrane (TBM) area of TBM-C4d positive staining case (Bar = 5 µm in **C**; Bar = 1 µm in **D**).

Of the 58 pediatric LN patients, PTC-C4d staining was seen in 34 cases (58.62%). PTC-C4d staining showed focal or diffuse granular deposition along the PTCs (Figures 3A–C). The semiquantitative scores of PTC-C4d staining revealed that a score of 2 was present in 12 patients (20.68%), a score of 1 was in 22 patients (37.97%), and 24 patients (41.37%) showed no PTC-C4d staining (a score of 0). In PTC-C4d positive staining LN cases, C4d co-deposition with immune complexes (IgG, IgA and IgM) along PTC was not detected by direct immunoﬂuorescence technique. Under transmission electron microscope, no any electron-dense deposits were observed in the PTC area (Figures 4A,B).

Clinically, compared to the PTC-C4d-negative patients, those with a PTC-C4d score of 1 or 2 showed significantly higher levels of serum creatinine and blood urea nitrogen, but lower levels of serum complements C3 and C4 (*P < *0.05). Meanwhile, there was no statistically significant difference between the patients with different PTC-C4d scores in terms of the average age, proteinuria, hematuria, or proportions with serum anti-dsDNA detection and hypertension (Table 4). Additionally, significantly higher SLEDAI values for LN patients were related to the patients with PTC-C4d scores of 2 or 1 (Table 4; score 2: 20.83 ± 3.83; score 1: 21.95 ± 2.8; negative: 14.7 ± 4.9; *P < *0.001). However, the SLEDAI values between those with a PTC-C4d score of 2 vs. 1 showed no statistical difference. Concerning the renal pathological AI of the renal biopsy, the patients with a PTC-C4d score of 2 or 1 had significantly greater values than those with PTC-C4d-negative results (score 2: 12.31 ± 2.59; score 1: 12.08 ± 4.48; negative: 7.87 ± 3.26; *P < *0.01). Similarly, there was no statistical difference in the renal pathological AI values between those with a PTC-C4d score of 2 vs. 1 (Table 4). Regarding the classification, 31 of the 34 PTC-C4d-positive cases were grouped into class IV (30 in class IV and 1 in class IV + V), whereas 3 were class III. The PTC-C4d-positive patients had a higher proportion of class IV LN compared to the PTC-C4d-negative patients (88.23%, 30 of 34 cases vs. 62.50%, 15 of 24 cases, *P < *0.05, Table 3). These results suggest that PTC-C4d positivity is a potential biomarker of disease activity and severity in pediatric SLE patients with LN.

**Table 4:** Clinical data and activity Index of renal injury according to the PTC-C4d staining score.

Characteristic	PTC-C4d score			*P*-value
	Score of 0 (*n *= 24)	Score of 1 (*n *= 22)	Score of 2 (*n *= 12)	
Age (years)	11.58 ± 2.78	11.77 ± 2.93	12.03 ± 4.48	0.9251
Proteinuria (g/24 h)	2.64 ± 2.24	3.05 ± 2.35	3.30 ± 2.84	0.7123
Hematuria (RBC/HPF)	175.67 ± 293.9	117.22 ± 184.41	176.70 ± 188.56	0.6592
Serum creatinine (µmol/L)	55.64 ± 23.38	81.18 ± 42.29	84.80 ± 30.05	0.0165*
Blood urea nitrogen (mmol/L)	6.52 ± 3.53	10.09 ± 5.37	10.35 ± 4.17	0.012*
Serum C3 (g/L)	0.544 ± 0.234	0.326 ± 0.167	0.240 ± 0.080	<0.0001**
Serum C4 (g/L)	0.068 ± 0.036	0.047 ± 0.021	0.031 ± 0.010	0.0008^#^
Anti-dsDNA, *n* (%)	20 (83.33%)	22 (100%)	12 (100%)	Not applicable
Hypertension, *n* (%)	5 (20.83%)	8 (36.66%)	4 (33.33%)	0.4833
SLEDAI	14.7 ± 4.9	21.95 ± 2.8	20.83 ± 3.83	<0.0001^##^
Activity index	7.87 ± 3.26	12.31 ± 2.59	12.08 ± 4.48	<0.0001*^#^

Data are expressed as the mean ± sd except for hypertension and anti-dsDNA.

* *P < *0.05 (score of 0 vs. score of 1; score of 0 vs. score of 2); *P > *0.05 (score of 1 vs. score of 2).

** *P < *0.001 (score of 0 vs. score of 1; score of 0 vs. score of 2); *P > *0.05 (score of 1 vs. score of 2).

^#^ *P < *0.001 (score of 0 vs. score of 1); *P < *0.05 (score of 0 vs. score of 2); *P > *0.05 (score of 1 vs. score of 2).

^##^ *P < *0.001 (score of 0 vs. score of 1; score of 0 vs. score of 2); *P > *0.05 (score of 1 vs. score of 2).

^#^ *P < *0.001 (score of 0 vs. score of 1); *P < *0.01 (score of 0 vs. score of 2); *P > *0.05 (score of 1 vs. score of 2).

### TBM-C4d is associated with the hypertension levels in pediatric LN patients

In addition to the glomeruli and PTCs, 11 (18.96%) pediatric LN patients harnessed C4d positivity on the tubular basement membrane (TBM). On the TBM, there were localized small granular deposits, which are characteristic of TBM-C4d staining (Figure 3D). In TBM-C4d positive staining LN cases, no immune complexes co-deposited with C4d were detected by direct immunoﬂuorescence and no any electron-dense deposits were observed in the TBM area under transmission electron microscope (Figures 4C,D). Regarding the average patient age, proteinuria, serum creatinine, blood urea nitrogen, hematuria, levels of serum complements, proportions of serum anti-dsDNA detection, SLEDAI, renal pathological AI of the renal biopsy, and pathological classes of LN, there was no obvious difference between the patients with TBM-C4d-positive staining and those with TBM-C4d-negative results (Tables 2, 3). However, the TBM-C4d-positive individuals had a considerably greater proportion of hypertension compared to the TBM-C4d-negative patients (63.63%, 7 of 11 cases vs. 21.27%, 10 of 47 cases, *P < *0.01, Table 2).

## Discussion

In this study, we focused on C4d detection among pediatric-onset LN cases. For the first time, we identified that G-C4d, PTC-C4d, and TMB-C4d were correlated with proteinuria, disease activity and severity, and hypertension, respectively, in the patients. These data suggest that renal C4d is a potential biomarker for disease activity and severity in pediatric LN patients.

According to the available findings from the adult-onset SLE population, Kim et al. pioneered the investigation into whether C4d deposition would be a useful marker for LN activity in 2003 (23). In their analysis of 21 LN patients, they noted that G-C4d deposition, whose primary site was the glomerular capillary loop, was not correlated with the renal pathological AI of biopsy specimens, proteinuria of LN patients, serum creatinine, or the levels of serum C3 and C4. Some of their observations were corroborated by subsequent studies (14, 24, 25). For example, Batal et al. found that G-C4d staining, which was detected in 92.5% of cases, was not correlated with LN renal pathological AI or CI (24). Moreover, a retrospective analysis of 20 LN patients by Kim et al. (25) revealed that G-C4d deposition is not related with the SLEDAI, renal pathological AI and CI, proteinuria, or levels of serum C3 and C4 in LN patients. However, some additional investigations revealed different results (26–29). Only 58% (14 of 24 patients) of LN patients had G-C4d staining in a study by Sahin et al. (28), in which the renal pathological AI score of renal injuries >12 and proteinuria showed higher proportions in G-C4d-positive patients. In a larger prospective study of 155 LN patients, the intensity of glomerular C4d staining was associated with the renal AI, serum C3 level, and 24-h urine protein level (29). Our current findings were consistent with previous studies to some extent. Similar to the former investigations (23, 25), we revealed that neither the renal pathological AI of the biopsy nor the SLEDAI of the LN patients was correlated with the intensity of G-C4d deposition. Meanwhile, our results demonstrated that high levels of G-C4d were associated with severe proteinuria, which was documented by the latter studies (28, 29). Therefore, renal G-4d is involved not only in adult-onset LN but also in patients with pediatric LN.

Based on a study by Kim et al. in 2003, G-C4d deposition, which does not correlate with the disease activity of LN, may merely reflect *in-situ* activation of the classical pathway by immune complex deposition (23). Such a notion was confirmed by Batal et al. (24) and our current findings, implying that the intense C4d deposition in the glomerulus LN patients, which does not associate with the disease activity of LN, may be the result of stronger or more activation of the classical pathway *in situ*. Furthermore, C4d deposition may have an impact on the glomerular filtration barrier and lead to severe proteinuria in LN patients.

Regarding PTC-C4d staining, Li et al. (16) and Drachenber et al. (14) detected PTC-C4d in only 6.8% (31 of 455 cases) and 8.8% (6 of 68 cases) of LN patients, respectively. Moreover, Yadav et al. (30) reported that PTC-C4d was not detected in LN patients. Nevertheless, the frequency of PTC-C4d detection was up to 86% and 77.3%, respectively, in research by Gonzalo et al. (31) and Allam et al. (15), respectively. Some researchers (15) have interpreted that such a discrepancy may be caused by the different detection techniques used, that both immunohistochemistry on parafﬁn sections and indirect immunoﬂuorescence on frozen tissue sections were employed for C4d staining. In our study, the frequency of PTC-C4d detection was 58.62% by immunohistochemistry on parafﬁn sections, which is consistent with the investigations using the same technique (15, 31). One of the possible reasons that the PTC-C4d detection frequency was higher on parafﬁn sections is that focal and minimal PTC-C4d-positive staining on parafﬁn sections is easier to read compared to that on frozen tissue sections. In our investigation, the PTC-C4d staining features revealed granular C4d deposition along the PTCs, which is in agreement with the findings of Allam et al. (15) and Li et al. (16). In addition, Li et al. (16) have reported that PTC-C4d-positive LN patients had higher renal pathological AI and SLEDAI scores as well as lower renal CI of the biopsy and levels of the complement components C3 and C4, which is in accordance with our data. Besides, our study also showed that the PTC-C4d-positive patients had a higher proportion of class IV LN compared to the PTC-C4d-negative patients, which has not been reported previously in any adult-onset SLE population study. These observations suggest that pediatric-onset LN patients have a greater disease activity or severity than adult-onset patients, implying that PTC-C4d staining is a more sensitive biomarker of pediatric-onset LN. Considering that the complement split product C4d can covalently bind to endothelial surfaces and basement membranes via a thioester moiety upon activation (13), our PTC-C4d staining results are reasonable. Moreover, PTC-C4d staining has been identified in renal biopsies with scleroderma renal crisis and primary Sjögren's syndrome (10, 32), suggesting that PTC-C4d staining may reflect the humoral immunity activity status of patients. In other words, PTC-C4d-positive staining indicated active, severe renal disease of LN and reflected active disease of SLE with LN.

In our study, 18.96% (11 of 58) of pediatric LN patients possessed TBM-C4d granular deposition. A similar finding has been reported by Batal et al. (24), who found that 3 of 15 LN cases had minimal or focal TBM-C4d deposition. Additionally, a higher proportion of patients with hypertension was observed in the TBM-C4d-positive patients compared to the TBM-C4d-negative patients in the current study. Further study is required to determine whether TBM-C4d-positive deposition is related to the poor outcomes of pediatric LN patients.

There are certain restrictions to this study. First, it was only a single-center retrospective investigation. In addition, the sample size was insufficient. Therefore, we look forward to performing a further prospective study with a much larger sample size to explore the significance of renal C4d deposition in pediatric LN.

In conclusion, G-4d, PTC-C4d, and TMB-C4d were associated with proteinuria, disease activity and severity, and hypertension, respectively, in pediatric LN patients. Our data suggest that renal C4d is a potential biomarker of disease activity and severity in pediatric LN, shedding light on the development of novel diagnostic and therapeutic methods for pediatric LN patients.

## Data Availability

The raw data supporting the conclusions of this article will be made available by the authors, without undue reservation.
